# Interferon-Inducible Oligoadenylate Synthetase-Like Protein Acts as an Antiviral Effector against Classical Swine Fever Virus via the MDA5-Mediated Type I Interferon-Signaling Pathway

**DOI:** 10.1128/JVI.01514-16

**Published:** 2017-05-12

**Authors:** Lian-Feng Li, Jiahui Yu, Yuexiu Zhang, Qian Yang, Yongfeng Li, Lingkai Zhang, Jinghan Wang, Su Li, Yuzi Luo, Yuan Sun, Hua-Ji Qiu

**Affiliations:** State Key Laboratory of Veterinary Biotechnology, Harbin Veterinary Research Institute, Chinese Academy of Agricultural Sciences, Harbin, China; Washington University School of Medicine

**Keywords:** Classical swine fever virus, interferon-stimulated genes, 2′-5′-oligoadenylate synthetase-like protein, antiviral activity, porcine MDA5, type I interferon

## Abstract

Classical swine fever virus (CSFV) is the causative agent of classical swine fever (CSF), which poses a serious threat to the global pig industry. Interferons (IFNs) and IFN-stimulated genes (ISGs) play a key role in host antiviral defense. We have previously screened the porcine 2′-5′-oligoadenylate synthetase-like protein (pOASL) as a potential anti-CSFV ISG using a reporter CSFV. This study aimed to clarify the underlying antiviral mechanism of pOASL against CSFV. We confirmed that CSFV replication was significantly suppressed in lentivirus-delivered, pOASL-overexpressing PK-15 cells, whereas silencing the expression of endogenous pOASL by small interfering RNAs markedly enhanced CSFV growth. In addition, the transcriptional level of pOASL was upregulated both *in vitro* and *in vivo* upon CSFV infection. Interestingly, the anti-CSFV effects of pOASL are independent of the canonical RNase L pathway but depend on the activation of the type I IFN response. Glutathione *S*-transferase pulldown and coimmunoprecipitation assays revealed that pOASL interacts with MDA5, a double-stranded RNA sensor, and further enhances MDA5-mediated type I IFN signaling. Moreover, we showed that pOASL exerts anti-CSFV effects in an MDA5-dependent manner. In conclusion, pOASL suppresses CSFV replication via the MDA5-mediated type I IFN-signaling pathway.

**IMPORTANCE** The host innate immune response plays an important role in mounting the initial resistance to viral infection. Here, we identify the porcine 2′-5′-oligoadenylate synthetase-like protein (pOASL) as an interferon (IFN)-stimulated gene (ISG) against classical swine fever virus (CSFV). We demonstrate that the anti-CSFV effects of pOASL depend on the activation of type I IFN response. In addition, we show that pOASL, as an MDA5-interacting protein, is a coactivator of MDA5-mediated IFN induction to exert anti-CSFV actions. This work will be beneficial to the development of novel anti-CSFV strategies by targeting pOASL.

## INTRODUCTION

Classical swine fever virus (CSFV) is the etiological agent of classical swine fever (CSF), which is an economically important viral disease of domestic pigs and wild boar worldwide. CSFV is a member of the genus Pestivirus, belonging to the family Flaviviridae (1, 2). The virus contains a single-stranded, positive-sense RNA genome of approximately 12.3 kb (3, 4). The viral genome comprises a single large open reading frame that encodes a polyprotein precursor of approximately 3,898 amino acids (aa) that is further proteolytically cleaved into 12 polypeptides comprised of four structural (C, E^rns^, E1, and E2) and eight nonstructural (N^pro^, p7, NS2, NS3, NS4A, NS4B, NS5A, and NS5B) proteins (5, 6).

Initiation of innate immunity in response to viral infections relies on sensing of viral nucleic acids by retinoic acid-inducible gene I (RIG-I)-like receptors (RLR) in the cytoplasm (7). Upon binding to viral RNAs, RIG-I or melanoma differentiation-associated gene 5 (MDA5) initiates a signaling cascade to induce the production of type I interferons (IFNs) (8, 9). IFNs exhibit pleiotropic effects through inducing transcription of hundreds of IFN-stimulated genes (ISGs) (9–12).

The 2′-5′-oligoadenylate synthetases (OASs) are IFN-induced antiviral enzymes. The human OAS family consists of four members, i.e., OAS1 to OAS3 and OAS-like protein (OASL) (10). Among them, OAS1 to OAS3 contain one, two, and three basic OAS units, respectively. The porcine OAS family consists of OAS1, OAS2, and OASL (13).

Members of the OAS family exert antiviral activity via the canonical OAS/RNase L-dependent or -independent antiviral pathway. The OAS/RNase L pathway has been studied extensively (10, 14). Activation of RNase L by the OAS family requires the OAS oligomerization unit and the processivity of synthesizing di-, tri-, and tetrameric 2′-5′-oligoadenylates (2-5A), which in turn bind and activate RNase L. Upon binding to 2-5A, RNase L dimerizes and degrades cellular and viral RNAs, resulting in the reduction of protein synthesis (15, 16). Moreover, some studies indicate that some members of the OAS family exhibit antiviral activity via several RNase L-independent pathways (12, 17, 18). For instance, human OASL (hOASL) exerts antiviral effects by enhancing RIG-I-mediated signaling by mimicking polyubiquitin (12).

In a previous study, we screened porcine OASL (pOASL) as a candidate anti-CSFV ISG (19). Several studies have demonstrated that hOASL has an N-terminal OAS-like domain without 2′-5′ OAS activity (12, 20, 21). Moreover, pOASL also contains only one OAS-like domain, lacking two C-terminal tandem ubiquitin-like domains (13). However, whether pOASL exhibits 2′-5′ OAS activity and how it exerts anti-CSFV actions remain unclear.

This study attempted to elucidate the role of pOASL in enhancing MDA5-mediated signaling and to clarify the mechanism by which pOASL exerts anti-CSFV activity. We have revealed a model whereby pOASL, induced upon CSFV infection, binds to MDA5 and functions as an anti-CSFV ISG via the MDA5-mediated type I IFN-signaling pathway.

## RESULTS

### pOASL inhibits CSFV replication.

**FIG 1:**
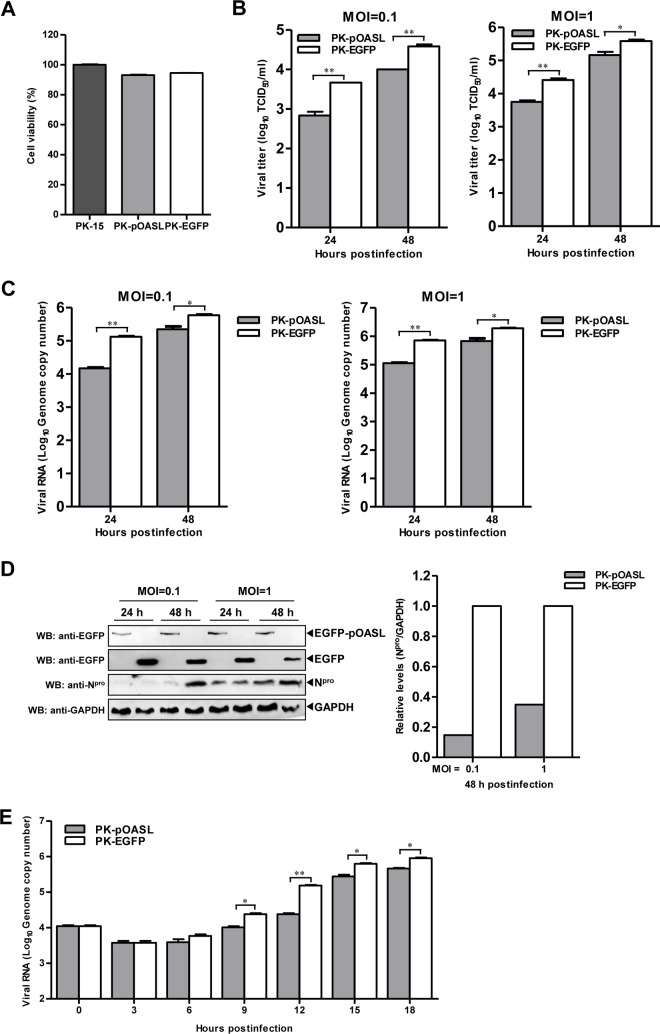
pOASL inhibits CSFV growth. (A) Viability of cell lines stably overexpressing pOASL. (B to D) Influence of overexpression of pOASL on CSFV replication. (B) PK-pOASL and PK-EGFP cells were infected with the Shimen strain of CSFV at MOI of 0.1 and 1 for 24 and 48 h. Viral titers in the harvested supernatants were titrated and determined. (C) The genomic RNA copy numbers of CSFV in the supernatants were quantified using real-time reverse transcription-PCR. (D) Expression of N^pro^ in the cell lysates was examined by Western blotting (WB) using a rabbit anti-N^pro^ PAb (1:500). GAPDH (glyceraldehyde-3-phosphate dehydrogenase) was included as an internal reference control. N^pro^ expression in the cell lysates was evaluated using Odyssey application software, version 3.0 (YQ09-0052; Li-Cor, USA). (E) Anti-CSFV activity of pOASL at early times after viral infection. PK-pOASL or PK-EGFP cells were infected with CSFV at a multiplicity of infection of 1 and harvested at 0, 3, 6, 9, 12, 15, and 18 h postinfection. The intracellular genomic copy numbers of CSFV were quantified by real-time RT-PCR. Each sample was run in triplicate. The error bars represent standard deviations. *, *P* < 0.05; **, *P* < 0.01.

To explore the antiviral activity of pOASL against CSFV, we established PK-pOASL and PK-EGFP cells stably expressing pOASL or enhanced green fluorescent protein (EGFP), respectively. The cell growth and viability of PK-pOASL and PK-EGFP cells were indistinguishable from those of the parent PK-15 cells (Fig. 1A). We have demonstrated that overexpression of pOASL inhibits rCSFV-Fluc replication (19). The anti-CSFV activity of pOASL was examined in PK-pOASL and PK-EGFP cells upon infection with the parental CSFV strain Shimen at multiplicities of infection (MOI) of 0.1 and 1. The extracellular titers of the progeny virus (Fig. 1B), the number of viral genomic copies (Fig. 1C), and the intracellular expression level of the N^pro^ protein (Fig. 1D) were significantly reduced in PK-pOASL cells compared to those in PK-EGFP cells at 24 or 48 h postinfection (hpi). However, the suppression of CSFV replication by OASL is different between MOI of 0.1 and 1. Considering that pOASL exerts antiviral effects against CSFV, we tried to investigate the antiviral kinetics of pOASL against CSFV replication. The intracellular viral genomic copy numbers of CSFV in infected cells were quantified at different time points postinfection. The results showed that the inhibition of CSFV at an MOI of 1 by pOASL reached a peak at 12 hpi and decreased thereafter (Fig. 1E).

**FIG 2:**
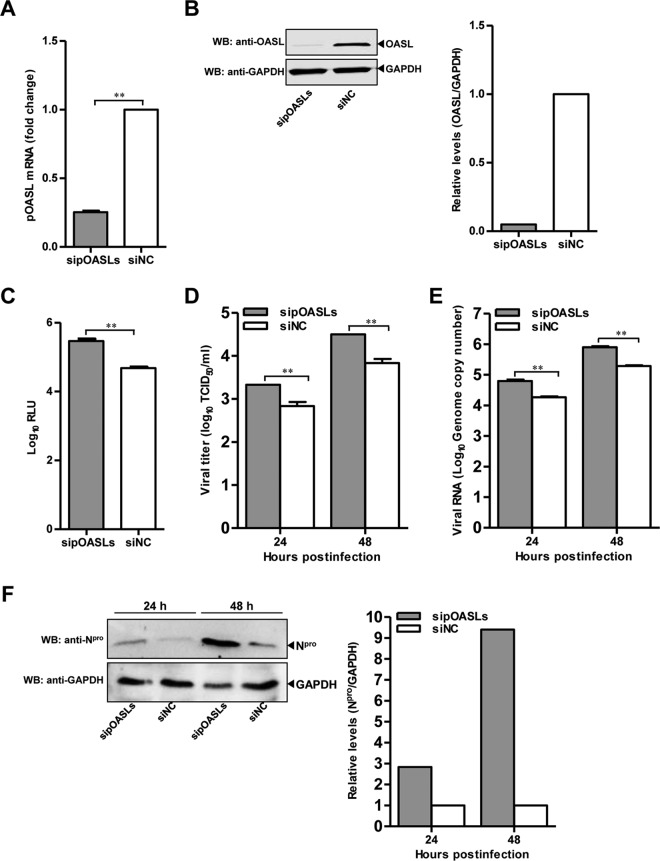
Knockdown of pOASL enhances CSFV growth. (A and B) Efficiency of knockdown of pOASL by siRNAs. (A) PK-15 cells transfected with a mixture of siRNAs (sipOASLs) targeting three regions of pOASL or siNC were collected at 36 hpt. The knockdown efficiency of pOASL was assessed by qRT-PCR. (B) For Western blotting analysis, PK-15 cells were initially pretreated with 0.1 μg of IFN-β (catalog no. RP0011S-005; Kingfisher) for 12 h and transfected with sipOASLs or siNC. At 36 hpt, pOASL and GAPDH were probed using a rabbit anti-OASL polyclonal antibody (catalog no. SAB2101671; Sigma; 1:500) and a mouse anti-GAPDH monoclonal antibody (1:1,000), respectively. (C) Influence of knockdown of pOASL on rCSFV-Fluc replication. PK-15 cells pretreated with sipOASLs or siNC (0.2 μM) for 36 h were infected with rCSFV-Fluc at an MOI of 0.1 for 48 h, and luciferase activities were measured using a luciferase reporter assay (Promega) and expressed in relative light units (RLU). (D to F) Effects of knockdown of pOASL on CSFV Shimen replication. PK-15 cells pretreated with sipOASLs or siNC (0.2 μM) for 36 h were infected with CSFV Shimen at an MOI of 0.1 for 24 and 48 h. (D) The extracellular viral titers were determined and expressed as TCID_50_/ml . (E) The supernatants were collected and the CSFV genomic copy numbers assessed using qRT-PCR . (F) The expression levels of N^pro^ and GAPDH were analyzed by Western blotting using a rabbit anti-N^pro^ polyclonal antibody (1:500) and a mouse anti-GAPDH monoclonal antibody, respectively. Each sample was run in triplicate. The error bars represent standard deviations. **, *P* < 0.01.

To further examine the antiviral effects of pOASL on CSFV, three specific small interfering RNAs (siRNAs) were used to silence the expression of endogenous pOASL in PK-15 cells, resulting in efficient reduction of pOASL expression (Fig. 2A and B). Subsequently, the replication efficiency of rCSFV-Fluc or Shimen was assessed upon silencing pOASL. Compared with the nontargeting control siRNA (siNC)-transfected control cells, the intracellular firefly luciferase (Fluc) activities and the expression of the N^pro^ protein were increased in PK-15 cells transfected with a mixture of three siRNAs targeting pOASL (sipOASLs) (Fig. 2C and F). Similarly, the extracellular viral genomic copy numbers and viral titers were increased significantly in these cells (Fig. 2E and D). The results demonstrate that knockdown of pOASL promotes CSFV replication.

### pOASL is activated upon CSFV infection.

**FIG 3:**
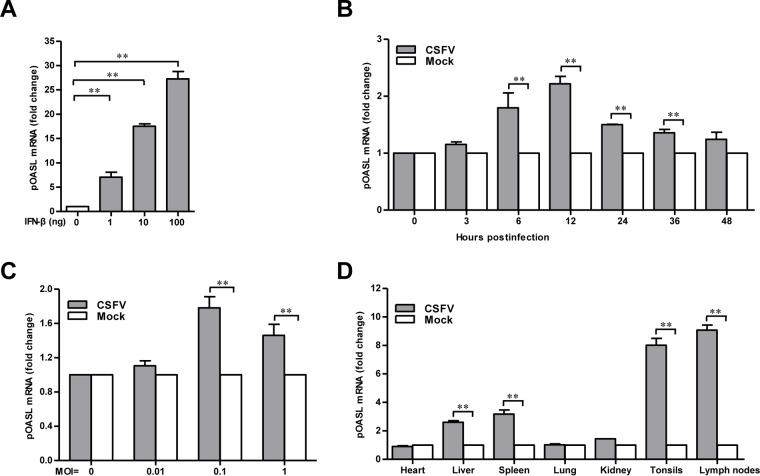
pOASL is upregulated upon CSFV infection. (A) Expression of pOASL in PK-15 cells treated with IFN-β (catalog no. RP0011S-005; Kingfisher). The expression of pOASL was examined by qRT-PCR in IFN-β-treated PK-15 cells. (B and C) Expression of pOASL in CSFV-infected PK-15 cells. PK-15 cells were infected with the Shimen strain of CSFV at an MOI of 0.1 and tested for the expression of pOASL at 0, 3, 6, 12, 24, and 48 hpi (B) or infected at MOI of 0, 0.01, 0.1, and 1 and tested at 6 hpi (C) by qRT-PCR. (D) Expression of pOASL in CSFV-infected pigs. Three healthy pigs negative for CSFV were inoculated with 10^5^ TCID_50_ CSFV Shimen. Various organs (heart, liver, spleen, lung, kidney, tonsils, and lymph nodes) were collected at 3 days postinoculation. The expression of pOASL in the different organs of the infected pigs was assessed by qRT-PCR. Each sample was run in triplicate. The error bars represent standard deviations. **, *P* < 0.01.

Since pOASL is able to inhibit CSFV replication, we attempted to examine the effects of CSFV infection on the expression of pOASL. PK-15 cells were infected with Shimen, and the transcription of pOASL was determined by real-time quantitative reverse transcription-PCR (qRT-PCR). As a control, PK-15 cells were treated with different doses of beta interferon (IFN-β). In accord with predictions, IFN-β activated the expression of pOASL in a dose-dependent manner (Fig. 3A). Similarly, the transcription level of pOASL was increased in the PK-15 cells infected with Shimen at an MOI of 0.1 at 3, 6, 12, 24, 36, and 48 hpi (Fig. 3B), as well as at MOI of 0.1 and 1 at 6 hpi (Fig. 3C). However, the expression of pOASL was lower at 48 hpi than at 36 hpi at an MOI of 0.1 and lower at an MOI of 1 than at an MOI of 0.1 at 6 hpi. We also showed that pOASL mRNA was expressed in all tested CSFV-infected organs, including heart, liver, spleen, lung, kidney, tonsils, and lymph nodes. High-level expression of pOASL was detected in liver, spleen, tonsils, and lymph nodes (Fig. 3D).

### The anti-CSFV effects of pOASL are independent of RNase L.

**FIG 4:**
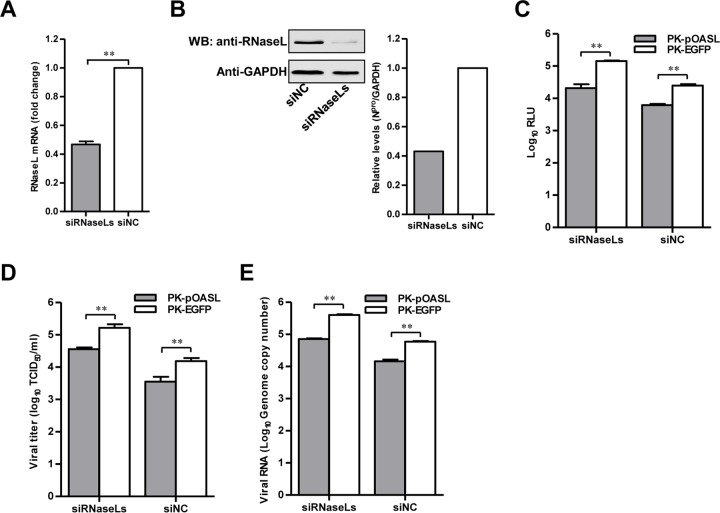
The anti-CSFV effects of pOASL are independent of the RNase L pathway. (A and B) Knockdown efficiency of RNase L expression by siRNAs. (A) PK-15 cells transfected with a mixture of three siRNAs (sipRNaseL-400, -599, and -750) targeting different regions of RNase L or siNC were harvested at 36 hpt. The knockdown efficiency of RNase L was checked by qRT-PCR. (B) For Western blotting, PK-15 cells were pretreated with 0.1 μg of IFN-β (catalog no. RP0011S-005; Kingfisher) for 12 h and transfected with sipRNaseLs or siNC. At 36 hpt, RNase L and GAPDH were probed using a rabbit anti-RNase L polyclonal antibody (catalog no. ab191392; Abcam; 1:500) and a mouse anti-GAPDH monoclonal antibody (1:1,000), respectively. (C) Influence of knockdown of RNase L on antiviral activity of pOASL against rCSFV-Fluc infection. PK-pOASL or PK-EGFP cells pretreated with siRNaseLs or siNC (0.2 μM) for 36 h were inoculated with rCSFV-Fluc at an MOI of 0.1 for 48 h, and luciferase activities were assayed using the luciferase reporter assay system (Promega) and expressed as RLU. (D and E) Effect of silencing RNase L on the antiviral activity of pOASL against CSFV Shimen. PK-pOASL or PK-EGFP cells were infected with the Shimen strain at an MOI of 0.1 for 48 h after pretreatment with siRNaseLs or siNC (0.2 μM) for 36 h. The viral titers were determined in the harvested supernatants at 48 h postinfection and presented as TCID_50_ per milliliter (D), and the CSFV genomic RNA copy numbers were determined by using qRT-PCR (E). Each sample was run in triplicate. The bars represent standard deviations. **, *P* < 0.01.

OAS are able to synthesize 2-5A, which leads to RNA degradation by activating RNase L (22). Considering that pOASL has an N-terminal OAS-like domain, we speculated that its antiviral activity likely depends on RNase L. We knocked down the RNase L expression in PK-15 cells using a mixture of three siRNAs targeting RNaseL (siRNaseLs) (Fig. 4A and B). In both PK-EGFP and PK-pOASL cells, knockdown of RNase L resulted in increased luciferase activities (Fig. 4C), extracellular viral titers (Fig. 4D), and viral genomic copy numbers (Fig. 4E) compared to those in siNC-transfected cells after infection with rCSFV-Fluc or Shimen. When RNase L was silenced, however, pOASL still exerted anti-CSFV actions, as demonstrated by lower viral genome RNA levels and virus yields in PK-pOASL than in PK-EGFP cells. These data suggested that the anti-CSFV activity of pOASL is independent of the RNase L pathway.

### pOASL enhances the type I IFN response.

**FIG 5:**
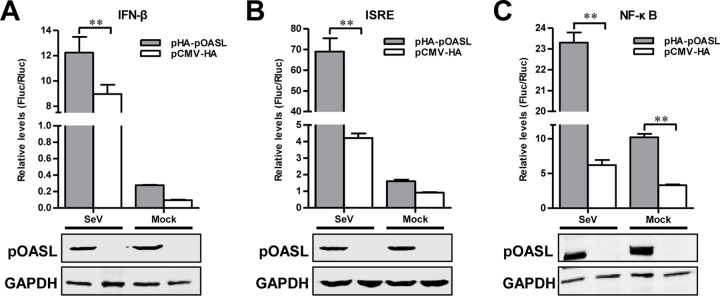
pOASL enhances type I IFN response. HEK293T cells were cotransfected with pHA-pOASL or pCMV-HA plus pIFN-β-Fluc and pTK-Rluc (A), pISRE-Fluc and pTK-Rluc (B), or pNF-κB-Fluc and pTK-Rluc (C) for 24 h; treated with 20 HAU/ml SeV for 24 h or left untreated; and assayed for luciferase activities using a dual-luciferase reporter assay (Promega). pTK-Rluc was used as an internal reference. The protein levels of HA-pOASL in HEK293T cells were measured by Western blotting. GAPDH served as a loading control. Each sample was run in triplicate. The error bars represent standard deviations. **, *P* < 0.01.

The results presented above reveal that the anti-CSFV activity of pOASL is independent of the classical RNase L pathway. Hence, whether pOASL exerts antiviral actions by an alternative mechanism needs to be investigated. It has been documented that several ISGs exert antiviral effects via various signaling pathways (12, 23, 24). To define the signaling pathway affected by pOASL, we conducted a dual-luciferase reporter assay. The results indicated that pOASL increased the promoter activities of IFN-β (Fig. 5A), ISRE (Fig. 5B), and NF-κB (Fig. 5C), implying that pOASL can enhance the IFN-β, ISRE, and NF-κB pathways.

### pOASL interacts with the RNA sensor MDA5.

**FIG 6:**
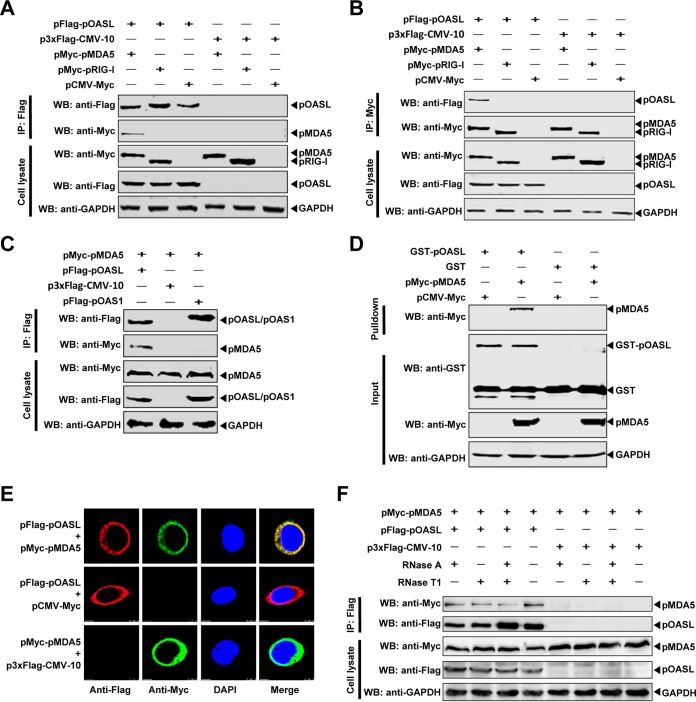
pOASL interacts with the MDA5 RNA sensor. (A to C) Co-IP assay. (A and B) HEK293T cells were cotransfected with pFlag-pOASL and either pMyc-pRIG-I, pMyc-pMDA5, or pCMV-Myc. The cells were harvested and subjected to co-IP assay using anti-Flag (A) and anti-Myc (B) MAbs. (C) HEK293T cells were cotransfected with pMyc-pMDA5 and either pFlag-pOASL, pFlag-pOAS1, or p3xFlag-CMV-10. The cell lysates were subjected to co-IP assay using anti-Flag MAb. The precipitated proteins were tested by Western blotting using the anti-Flag and anti-Myc antibodies. (D) GST pulldown assay. GST and GST-pOASL fused proteins expressed in E. coli BL21 were affinity purified with glutathione beads and incubated with the Myc-pMDA5 protein. The proteins bound to beads were analyzed by immunoblotting using a mouse anti-GST polyclonal antibody (1:2,000) and a mouse anti-Myc MAb (1:1,000). (E) Confocal assay. BHK-21 cells were cotransfected with pFlag-pOASL and pMyc-pMDA5 and subjected to confocal assay. (F) The pMDA5-pOASL interaction is independent of RNA. HEK293T cells were cotransfected with pFlag-pOASL and pMyc-pMDA5. The cell lysates were harvested and treated with RNases A and T1. Co-IP was performed using an anti-Flag MAb (1:1,000).

It has been reported that hOASL interacts with RIG-I and enhances RIG-I-mediated type I IFN signaling (12). CSFV has been verified to trigger the RIG-I- and MDA5-dependent signaling pathway (25). Indeed, our data also demonstrated that pOASL activates the type I IFN response (Fig. 5). Based on the above-mentioned results, we hypothesized that pOASL plays a role in RIG-I- or MDA5-mediated signaling. The interaction between pOASL and porcine RIG-I (pRIG-I) or MDA5 (pMDA5) was investigated using a coimmunoprecipitation (co-IP) assay. The results showed that the Flag-tagged pOASL interacted with the Myc-tagged pMDA5, but not with the Myc-tagged pRIG-I (Fig. 6A and B). Furthermore, the Myc-tagged pMDA5 was shown to coimmunoprecipitate with the Flag-tagged pOASL, but not the Flag-tagged pOAS1 (Fig. 6C). Glutathione *S*-transferase (GST) pulldown assay further confirmed that GST-pOASL, but not GST, interacted with pMDA5 (Fig. 6D). Furthermore, the pOASL-pMDA5 interaction was confirmed by confocal assay (Fig. 6E). To exclude the involvement of RNA in the pOASL-pMDA5 interaction, the cell lysates were treated with RNases A and T1, followed by co-IP. The results indicated that the interaction of pOASL and pMDA5 was independent of RNA (Fig. 6F).

### pOASL enhances the MDA5-mediated type I IFN response.

**FIG 7:**
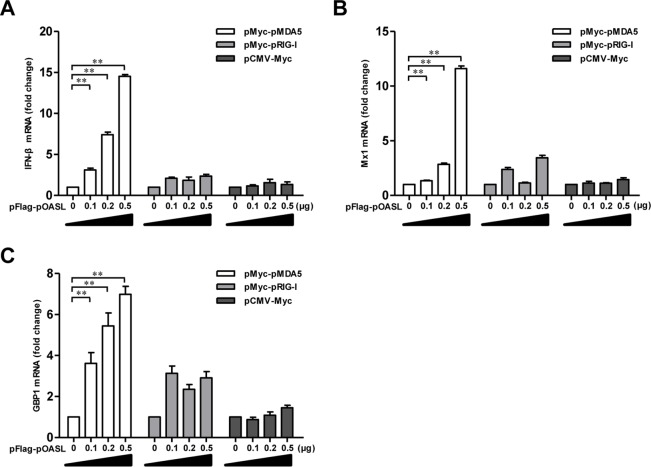
pOASL enhances the MDA5-mediated type I IFN-signaling pathway. pOASL upregulates the transcriptional levels of IFN-β and ISGs in PK-15 cells. PK-15 cells were cotransfected with pHA-OASL (0, 0.1, 0.2, or 0.5 μg) plus pMyc-pMDA5 (0.5 μg), pMyc-pRIG-I (0.5 μg), or pCMV-Myc (0.5 μg) for 24 h, and the transcriptional levels of IFN-β (A), Mx1 (B), and GBP1 (C) in the cells were determined by qRT-PCR assay. Each sample was run in triplicate. The error bars represent standard deviations. **, *P* < 0.01.

Because pOASL interacts with pMDA5 and activates the IFN-β signaling pathway, we endeavored to investigate whether the action of pOASL is mediated via the pMDA5 pathway. We tried to determine whether pOASL coexpressed with pMDA5 affects the transcription of IFN-β and ISGs in PK-15 cells. The results showed that the coexpression of pOASL with pMDA5, but not with pRIG-I, markedly upregulated the mRNA transcriptional levels of IFN-β, myxovirus resistance protein 1 (Mx1), and guanylate-binding protein 1 (GBP1) (Fig. 7A to C). Collectively, these studies support the idea that pOASL positively regulates pMDA5-mediated type I IFN signaling.

### Antiviral activity of pOASL is abolished in MDA5 knockdown cells.

**FIG 8:**
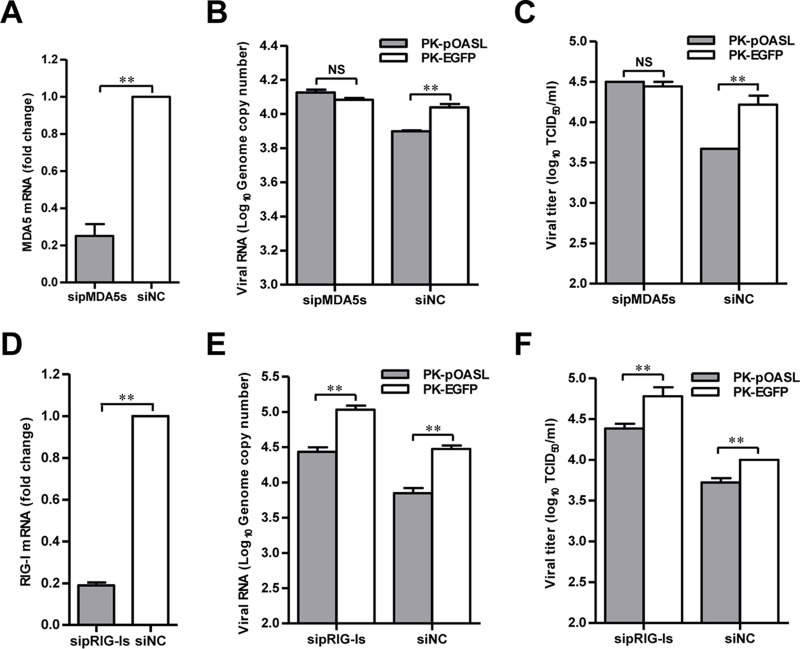
The antiviral activity of pOASL is dependent on MDA5. (A) Knockdown efficiency of pMDA5 by siRNAs. PK-15 cells were transfected with a mixture of three siRNAs targeting pMDA5 (sipMDA5s) to silence pMDA5 by targeting different regions or with control siRNA (siNC) and collected at 36 hpt. The efficiency of pMDA5 knockdown was examined by qRT-PCR. (B and C) Silencing pMDA5 expression reduces the antiviral activity of pOASL against CSFV. (B) PK-pOASL or PK-EGFP cells were transfected with sipMDA5s or siNC (0.2 μM) for 24 h and infected with CSFV at an MOI of 0.1 for 48 h. Total RNA was extracted from the harvested cells and quantified to evaluate CSFV replication by qRT-PCR. (C) The viral titers in the harvested supernatants were titrated and determined as TCID_50_ per milliliter. (D) Knockdown efficiency of pRIG-I expression by siRNAs. (E and F) Silencing pRIG-I expression does not affect the anti-CSFV activity of pOASL as assessed by viral genomic copy numbers (E) and viral titers (F). Each sample was run in triplicate. The error bars represent standard deviations. NS, not significant; **, *P* < 0.01.

MDA5 is one of the primary sensors of viral RNA for CSFV infection (25). In light of the fact that pOASL displayed antiviral activity against CSFV depending on pMDA5-mediated type I IFN signaling (Fig. 7), we silenced pMDA5 expression using siRNAs to further confirm the results. Marked reduction of pMDA5 expression was observed in the pMDA5-silenced cells (Fig. 8A). As expected, significant reduction in CSFV replication was observed in the siNC-transfected PK-pOASL cells. Upon silencing of pMDA5, however, pOASL almost lost its ability to inhibit CSFV replication in PK-pOASL cells, as assessed by the viral genomic copy numbers and the viral titers (Fig. 8B and C). In contrast, in the cells with knockdown of pRIG-I (Fig. 8D), the anti-CSFV activity of pOASL was not affected (Fig. 8E and F). These data indicate that the anti-CSFV activity of pOASL is dependent on pMDA5.

## DISCUSSION

In this study, we clarified the antiviral mechanism by which pOASL binds to pMDA5 and enhances pMDA5-mediated antiviral signaling. We showed that pOASL efficiently suppressed CSFV growth in PK-15 cells (Fig. 1 and 2). Interestingly, we found that pOASL expression was activated *in vitro* at an earlier phase after viral infection and then declined (Fig. 3) and that the anti-CSFV effects of pOASL depend on enhancement of the type I IFN response, but not on the canonical RNase L pathway (Fig. 4 and 5). Notably, we demonstrated that pOASL interacts with pMDA5 and enhances pMDA5-mediated type I IFN antiviral signaling to antagonize the replication of CSFV (Fig. 6 to 8). Taken together, these findings suggest that pOASL is an anti-CSFV ISG via the pMDA5-mediated type I IFN-signaling pathway.

The IFN-inducible OASs are important components of the antiviral activity of IFNs and are involved in other cellular processes, including cell growth, apoptosis, gene regulation, cell differentiation, RNA splicing, and DNA replication (26, 27). In humans, there are three functional *OAS* genes (*OAS1* to *OAS3*), including 8 to 10 *OAS* isoforms due to alternative mRNA splicing (28). In mice, in addition to *OAS2* and *OAS3*, there are eight *OAS1* genes (29, 30). The pig and cattle genomes contain three *OAS* genes, *OAS1*, *OAS2*, and *OASL*. The human *OASL* gene has one OAS unit and two ubiquitin-like repeats on the C terminus (31). While two tandemly duplicated *OASL* genes have been identified in the dog genome, only a single OASL ortholog has been found in the cattle or pig genome (26, 32). The porcine and bovine *OASL* genes contain a premature stop codon, resulting in truncated proteins that lack the typical C-terminal ubiquitin domains. Indeed, we demonstrated that the porcine *OASL* gene contains only a single OAS domain (33).

Viral infection usually upregulates the expression of ISGs, which inhibit viral replication. It has been demonstrated that both OAS1 and OASL mRNA levels peak around 10 h after influenza A virus infection and then decline, and the decline observed in virus-infected cells was probably due to the synthesis of viral inhibitors of the innate immune system, such as the influenza A virus NS1 protein (34, 35). Similarly, our data also showed that the mRNA level of pOASL peaked around 12 h after infection and then declined subsequently. Specifically, the mRNA level of pOASL was lower at 48 hpi than at 24 hpi at an MOI of 0.1 (Fig. 3B) and lower at an MOI of 1 than at an MOI of 0.1 (Fig. 3C) at 6 hpi. A possible explanation is that CSFV has the ability to escape the antiviral action of pOASL. It has been shown that OASL is rapidly induced upon viral infection through interferon regulatory factor 3 (IRF3), as well as IFN signaling (11, 36), and that CSFV N^pro^ interacts with IRF3 and induces its proteasomal degradation to prevent type I IFN induction (34, 37). Thus, high doses of CSFV may increase the chance of IRF3 degradation, leading to decreased expression of pOASL.

With hundreds of ISGs induced by IFN, we assume that any step of the viral life cycle (entry, uncoating, transcription, translation, assembly, or egress) could be targeted for inhibition (38). The OAS family are well-known molecules that regulate the early phase of viral infection (39), and the mouse Oas1b acts mainly on the replication stage of West Nile virus (WNV) (17). In this study, we investigated the antiviral kinetics of pOASL on CSFV replication. The data demonstrated that the viral genomic RNA copy numbers were decreased in PK-pOASL cells compared to those in PK-EGFP cells from 6 to 12 hpi (Fig. 1E). However, our experiments do not define the exact stage(s) of the CSFV life cycle that pOASL targets. Thus, we need to further investigate the exact stage of CSFV replication that pOASL targets using different methods in the future.

hOASL interacts with RIG-I and enhances RIG-I-mediated antiviral signaling (12). Similar to hOASL, mouse Oasl2 exhibits antiviral activity, and its loss results in the reduction of IFN signaling and enhances RNA virus replication (12). However, it has been demonstrated that mouse Oasl1 binds to the 5′ untranslated region (UTR) of IRF7 to inhibit IRF7 translation, thus reducing IFN induction and increasing viral replication (40). In this study, we showed that the interaction of pOASL with pMDA5 enhances the pMDA5-mediated type I IFN-signaling pathway, which increases IFN induction and efficiently inhibits CSFV replication.

The OAS proteins bind to virus-derived double-stranded RNA (dsRNA) and result in allosteric activation of OAS, giving rise to the synthesis of 2-5A from ATP, which activates RNase L. Upon 2-5A binding, RNase L dimerizes and degrades cellular, as well as viral, RNA in the cell, causing the inhibition of protein synthesis (14, 15). The OAS/RNase L system is a component of IFN-regulated innate antiviral immunity. Our study revealed that pOASL inhibits viral replication at the early stages of the CSFV life cycle. We also demonstrated that pOASL contains only a single N-terminal OAS-like domain and lacks C-terminal double ubiquitin domains. To investigate the sensitivity of CSFV to the pOASL antiviral pathway at the molecular level, we supposed that pOASL exerting an antiviral effect is associated with the OAS/RNase L system, based on sequence alignment. The results showed that RNase L knockdown by specific siRNAs did not result in an obvious change of CSFV replication. Thus, we conclude that pOASL exerts antiviral effects independently of the canonical RNase L pathway but depending on the noncanonical pathway.

It has been indicated that hOASL plays a critical role in resistance to HCV infection *in vivo* (41). The hOASL protein displays antiviral activity and activates RIG-I by mimicking polyubiquitin. HCV and CSFV are both single-stranded, positive-sense RNA viruses belonging to the family Flaviviridae. Hence, we suppose that pOASL exerts antiviral actions also depending on activation of the type I IFN-signaling pathway. Indeed, in this study, we revealed that pOASL could enhance the type I IFN-signaling pathway.

hOASL interacts and colocalizes with RIG-I and enhances RIG-I signaling via its C-terminal ubiquitin-like domain (12). Our study indicated that pOASL interacts with pMDA5, but not pRIG-I, and enhances pMDA5-mediated IFN induction. Our study reveals a mechanism by which pOASL exerts antiviral action via the MDA5-mediated IFN-signaling pathway.

In conclusion, we demonstrate that pOASL is an anti-CSFV ISG that depends on the MDA5-mediated type I IFN-signaling pathway.

## MATERIALS AND METHODS

### Cells and viruses.

PK-15 (a porcine kidney cell line) cells were cultured in Dulbecco's modified Eagle's medium (DMEM) (Gibco) containing 5% fetal calf serum (FCS) (Sigma-Aldrich) in 5% CO_2_ at 37°C. BHK-21 (a Syrian baby hamster kidney cell line) and HEK293T (a human embryonic kidney cell line) cells were cultured similarly in DMEM plus 10% FCS.

rCSFV-Fluc, a reporter CSFV harboring the Fluc gene (42), was used in the antiviral assays of pOASL. rCSFV-Fluc and the CSFV strain Shimen, the parent virus of rCSFV-Fluc, were propagated in PK-15 cells. Sendai virus (SeV) was propagated in 9-day-old specific-pathogen-free chicken embryos.

### Plasmid construction and cell transfection.

The *pOASL* gene (GenBank accession no. NM_001031790) was cloned into the vector pCMV-HA (Clontech), p3xFlag-CMV-10 (Sigma-Aldrich), or pFUGW (Addgene), creating pHA-pOASL, pFlag-pOASL, or pFUGW-pOASL. The primers for amplification of the *pOASL* gene are listed in Table 1.

**TABLE 1:** Primers used in this study

Primer	Sequence (5′–3′)	Use
HA-pOASL-F	CGGAATTCGGATGGAGCTATTTTACACCCCAG	Amplification of pOASL
HA-pOASL-R	GCGTCGACTCAGTCACAGCCTTTGGCTGAG	
Flag-pOASL-F	CGGAATTCAATGGAGCTATTTTACACC	Amplification of pOASL
Flag-pOASL-R	GAAGATCTTCAGTCACAGCCTTTGGC	
GST-pOASL-F	CCGGAATTCATGGAGCTATTTTACACCCCAG	Amplification of pOASL
GST-pOASL-R	GCGTCGACTCAGTCACAGCCTTTGGCTGAG	
EGFP-pOASL-F	ACAGGCCATTACGGCCATGGAGCTATTTTACACC	Amplification of pOASL
EGFP-pOASL-R	TACGGCCGAGGCGGCCTTATCAGTCACAGCCTTTGGC	
Myc-pMDA5-F	CCGGAATTCGGATGTCGTCGGATGGGTATTC	Amplification of pMDA5
Myc-pMDA5-R	CCGCTCGAGTCAGTCCTCATCACTAGAC	
Q-pMDA5-F	GAAGATGACAGTGATGACAATGG	qRT-PCR for detection of pMDA5
Q-pMDA5-R	ATTATTCCTCGTGCTGATTCCTC	
Q-pRIG-I-F	CAACACCAGCAAACAGCATCC	qRT-PCR for detection of pRIG-I
Q-pRIG-I-R	CCGAGGCAGTCAGTCCAATG	
Q-pOASL-F	GAAGAATGTGCAGGTGCTAGAGG	qRT-PCR for detection of pOASL
Q-pOASL-R	CCCTGGCAAGAGCATAGTGTC	
Q-IFN-β-F	GGCTGGAATGAAACCGTCAT	qRT-PCR for detection of IFN-β
Q-IFN-β-R	TCCAGGATTGTCTCCAGGTCA	
Q-Mx1-F	GAACGAAGAAGACGAATGGAAGG	qRT-PCR for detection of Mx1
Q-Mx1-R	GATGCCAGGAAGGTCTATGAGG	
Q-GBP1-F	GAAGGGTGACAACCAGAACGAC	qRT-PCR for detection of GBP1
Q-GBP1-R	AGGTTCCGACTTTGCCCTGATT	
Q-GAPDH-F	GAAGGTCGGAGTGAACGGATTT	qRT-PCR for detection of GAPDH
Q-GAPDH-R	TGGGTGGAATCATACTGGAACA	

HEK293T cells seeded in six-well plates (Corning) were transfected with 2 μg of pHA-pOASL and 2 μl of X-tremeGene HP DNA transfection reagent (catalog no. 06366236001; Roche) according to the manufacturer's instructions. At 6 h posttransfection (hpt), the medium was changed to fresh DMEM containing 10% FCS, and the cells were incubated for another 48 h for further assays.

### Establishment and identification of a cell line overexpressing pOASL.

To construct a stable cell line overexpressing pOASL, HEK293T cells grown in 10-cm cell dishes were cotransfected with 10.5 μg of pFUGW-pOASL or pFUGW-EGFP harboring the *egfp* gene (serving as a control), in combination with 7 μg of psPAX2 and 3.5 μg of pMD2.G, and the resulting recombinant lentiviruses, Lenti-pOASL and Lenti-EGFP, were harvested at 48 hpt from the supernatants of the transfected cells. Subsequently, to generate the stable cell lines PK-pOASL and PK-EGFP, PK-15 cells were transduced with the above-mentioned lentiviruses at an MOI of 1. The expression of EGFP-pOASL and EGFP in the cells was immunoblotted with a mouse anti-EGFP monoclonal antibody (MAb) (1:1,000; catalog no. A00185; GeneScript).

### Cell viability assay.

To evaluate the effects of ectopic expression on the growth of the cell lines, a cell viability assay was carried out using cell counting kit 8 (CCK-8) (catalog no. CK04; Dojindo) according to the manufacturer's instructions.

### IFA and virus titration.

CSFV was titrated based on indirect immunofluorescence assay (IFA) as described previously (19). Viral titers were calculated according to a previous report (43) and expressed as median tissue culture infective doses (TCID_50_)/ml.

### Real-time RT-PCR.

Total RNA was isolated from the CSFV-infected PK-15 cells or swine organs using TRIzol reagent (catalog no. 15596026; Invitrogen) and reverse transcribed into the cDNA using reverse transcriptase XL (avian myeloblastosis virus [AMV]) (catalog no. 2621; TaKaRa) according to the manufacturer's instructions. A qRT-PCR assay was performed to quantify the genomic RNA copy numbers of CSFV (44).

### RNA interference assay.

Three specific siRNAs targeting different regions of the *pOASL*, *pRNaseL*, or *pMDA5* gene and a nontargeting control siRNA (siNC) were commercially synthesized by GenePharma; the siRNAs are listed in Table 2. The siRNA transfection assay was performed as described previously (19). PK-15 cells (5 × 10^4^ cells/well) were plated in 24-well plates and transfected with 0.2 μM sipOASLs or siNC using X-tremeGene siRNA transfection reagent (catalog no. 4476093001; Roche) according to the manufacturer's instructions. At 36 hpt, the transfected cells were inoculated with rCSFV-Fluc or Shimen at an MOI of 0.1. After 2 h, the cell medium was replaced with fresh DMEM containing 2% FCS and incubated at 37°C. At 48 hpi, the supernatants and lysates of the cells were collected for further analysis.

**TABLE 2:** siRNAs used in this study

siRNA	Sequence	
	Sense (5′–3′)	Antisense (5′–3′)
sipOASLs		
sipOASL-479	CCGAGGACCUCGAUAACAUTT	AUGUUAUCGAGGUCCUCGGTT
sipOASL-905	CCUGGGAAGUGGGUACAAATT	UUUGUACCCACUUCCCAGGTT
sipOASL-1222	CCUUCCCACUACACUGCUUTT	AAGCAGUGUAGUGGGAAGGTT
sipRNaseLs		
siRNaseL-400	GGUAGAGUCGAAGCCUUAATT	UUAAGGCUUCGACUCUACCTT
siRNaseL-599	GCAGAAAUGCUUUGGUCUATT	UAGACCAAAGCAUUUCUGCTT
siRNaseL-750	GGAUUUGGUGCAGAUGCUUTT	AAGCAUCUGCACCAAAUCCTT
sipMDA5s		
siMDA5-1441	CCUCAGAUAUUGGGACUAATT	UUAGUCCCAAUAUCUGAGGTT
siMDA5-1848	CCGAAGGAUUGAUGCCUAUTT	AUAGGCAUCAAUCCUUCGGTT
siMDA5-2195	CCCAGUGGAUUACUGACAATT	UUGUCAGUAAUCCACUGGGTT
sipRIG-Is		
siRIG-I-946	GGUACAAAGUUGCAGGCATT	UGCCUGCAACUUUGUACCTT
siRIG-I-1126	GCAAACAGCAUCCUUAUAATT	UUAUAAGGAUGCUGUUUGCTT
siRIG-I-1477	CCAUAACUCUUGGAGGCUUTT	AAGCCUCCAAGAGUUAUGGTT
siNC		
Nontargeting control	UUCUCCGAACGUGUCACGUTT	ACGUGACACGUUCGGAGAATT

### Animal infection experiment.

To test the expression level of pOASL in CSFV-infected pigs, three pigs were left uninfected or infected with 10^5^ TCID_50_ Shimen. Various organs (heart, liver, spleen, lung, kidney, tonsils, and lymph nodes) were collected at 3 days postinoculation (45). The mRNA expression level of pOASL was evaluated by qRT-PCR as described previously (19).

### Dual luciferase reporter assay.

HEK293T cells in 24-well plates were transfected with pHA-pOASL or pCMV-HA (0.5 μg each), together with the promoter reporter plasmid pIFN-β-Fluc, pISRE-Fluc, or pNF-κB-Fluc (0.2 μg each) and the internal reference reporter plasmid expressing TK-Renilla luciferase (Rluc) (10 ng). At 24 hpt, the transfected cells were stimulated with SeV at 20 hemagglutinin units (HAU)/ml or left untreated for 24 h and assayed for reporter activities using the Dual-Luci assay kit (Promega). The luciferase activities are presented as Fluc/Rluc relative expression levels.

### Co-IP and Western blotting.

HEK293T cells were cotransfected with pFlag-pOASL and pMyc-pMDA5 or pMyc-pRIG-I expressing pMDA5 or pRIG-I (2 μg each). At 48 hpt, the cells were lysed with NP-40 lysis buffer (catalog no. P0013F; Beyotime) containing phenylmethanesulfonyl fluoride (PMSF) to a final concentration of 1 mM for 1 h at 4°C. To remove cellular debris, the lysates were centrifuged at 12,000 × *g* at 4°C for 25 min, and protein G-agarose (catalog no. 11243233001; Roche) was used to preclear the supernatants for 4 h at 4°C and then incubated with an anti-Flag MAb (catalog no. F1804; Sigma-Aldrich) or with an irrelevant isotype-matched MAb (catalog no. A00185; GeneScript) acting as a negative control for 5 h at 4°C. The immunoprecipitates were washed five times with the NP-40 lysis buffer and dissolved in phosphate-buffered saline (PBS) for Western blotting, as mentioned above.

### GST pulldown assay.

To produce GST-tagged pOASL protein, the *pOASL* gene was subcloned into the prokaryotic expression vector pGEX-6p-1 (GE Healthcare). The resulting recombinant plasmid, pGST-pOASL, was transformed to express the GST-tagged pOASL (GST-pOASL) in Escherichia coli BL21(DE3). GST-pOASL was purified as described previously (19). The GST pulldown assay was performed by incubating the GST-pOASL-bound resin with the Myc-tagged pMDA5 produced in HEK293T cells for 5 h at 4°C. Immunoblotting was performed to probe the pulled down proteins using a mouse anti-GST polyclonal antibody (PAb) (catalog no. AB101; Tiangen) and a mouse anti-Myc MAb (catalog no. SAB4700447; Sigma-Aldrich).

### Confocal imaging.

BHK-21 cells were cotransfected with pFlag-pOASL and pMyc-pMDA5 (2 μg each). At 36 hpt, the cells were immunostained with the respective antibodies and observed using a Leica SP2 confocal microscope (Leica Microsystems), as described previously (19).

### Statistical analysis.

All data were analyzed using SPSS 18.0 software. Differences were considered to be significant if an unadjusted *P* value was less than 0.05.

## References

[1] Lindenbach BD, Murray CL, Thiel HJ 2013 Flaviviridae, p 712–746. *In* Knipe DM, Howley PM, Cohen JI, Griffin DE, Lamb RA, Martin MA, Racaniello VR, Roizman B (ed), Fields virology, 6th ed, vol 2 Lippincott Williams & Wilkins, Philadelphia, PA.

[2] Pletnev A, Gould E, Heinz FX, Meyers G, Thiel HJ, Bukh J, Stiasny K, Collett MS, Becher P, Simmonds P, Rice CM, Monath TP 2011 Flaviviridae, p 1003–1020. *In* King AMQ, Adams MJ, Carstens EB, Lefkowitz EJ (ed), Virus taxonomy, 9th ed Academic Press, Oxford, United Kingdom.

[3] Rümenapf T, Meyers G, Stark R, Thiel HJ 1991 Molecular characterization of hog cholera virus. Arch Virol Suppl 3:7–18. doi:10.1007/978-3-7091-9153-8_2. 9210921

[4] Moormann RJ, Warmerdam PA, van der Meer B, Schaaper WM, Wensvoort G, Hulst MM 1990 Molecular cloning and nucleotide sequence of hog cholera virus strain Brescia and mapping of the genomic region encoding envelope protein E1. Virology 177:184–198. doi:10.1016/0042-6822(90)90472-4. 2162104

[5] Collett MS, Moennig V, Horzinek MC 1989 Recent advances in pestivirus research. J Gen Virol 70:253–266. doi:10.1099/0022-1317-70-2-253. 2471785

[6] Thiel HJ, Stark R, Weiland E, Rümenapf T, Meyers G 1991 Hog cholera virus: molecular composition of virions from a pestivirus. J Virol 65:4705–4712. 1870198 10.1128/jvi.65.9.4705-4712.1991 PMC248926

[7] Yoneyama M, Kikuchi M, Natsukawa T, Shinobu N, Imaizumi T, Miyagishi M, Taira K, Akira S, Fujita T 2004 The RNA helicase RIG-I has an essential function in double-stranded RNA-induced innate antiviral responses. Nat Immunol 5:730–737. doi:10.1038/ni1087. 15208624

[8] Loo YM, Gale M Jr 2011 Immune signaling by RIG-I-like receptors. Immunity 34:680–692. doi:10.1016/j.immuni.2011.05.003. 21616437 PMC3177755

[9] Yan N, Chen ZJ 2012 Intrinsic antiviral immunity. Nat Immunol 13:214–222. doi:10.1038/ni.2229. 22344284 PMC3549670

[10] Sadler AJ, Williams BR 2008 Interferon-inducible antiviral effectors. Nat Rev Immunol 8:559–568. doi:10.1038/nri2314. 18575461 PMC2522268

[11] Sarkar SN, Sen GC 2004 Novel functions of proteins encoded by viral stress-inducible genes. Pharmacol Ther 103:245–259. doi:10.1016/j.pharmthera.2004.07.007. 15464592

[12] Zhu J, Zhang Y, Ghosh A, Cuevas RA, Forero A, Dhar J, Ibsen MS, Schmid-Burgk JL, Schmidt T, Ganapathiraju MK, Fujita T, Hartmann R, Barik S, Hornung V, Coyne CB, Sarkar SN 2014 Antiviral activity of hOASL protein is mediated by enhancing signaling of the RIG-I RNA sensor. Immunity 40:936–948. doi:10.1016/j.immuni.2014.05.007. 24931123 PMC4101812

[13] Perelygin AA, Zharkikh AA, Scherbik SV, Brinton MA 2006 The mammalian 2′-5′ oligoadenylate synthetase gene family: evidence for concerted evolution of paralogous Oas1 genes in Rodentia and Artiodactyla. J Mol Evol 63:562–576. doi:10.1007/s00239-006-0073-3. 17024523

[14] Chakrabarti A, Jha BK, Silverman RH 2011 New insights into the role of RNase L in innate immunity. J Interferon Cytokine Res 31:49–57. doi:10.1089/jir.2010.0120. 21190483 PMC3021357

[15] Clemens MJ, Williams BR 1978 Inhibition of cell-free protein synthesis by pppA2′p5′A2′p5′A: a novel oligonucleotide synthesized by interferon-treated L cell extracts. Cell 13:565–572. doi:10.1016/0092-8674(78)90329-X. 657268

[16] Dong B, Silverman RH 1997 A bipartite model of 2-5A-dependent RNase L. J Biol Chem 272:22236–22242. doi:10.1074/jbc.272.35.22236. 9268370

[17] Kajaste-Rudnitski A, Mashimo T, Frenkiel MP, Guénet JL, Lucas M, Desprès P 2006 The 2′,5′-oligoadenylate synthetase 1b is a potent inhibitor of West Nile virus replication inside infected cells. J Biol Chem 281:4624–4637. doi:10.1074/jbc.M508649200. 16371364

[18] Kristiansen H, Scherer CA, McVean M, Iadonato SP, Vends S, Thavachelvam K, Steffensen TB, Horan KA, Kuri T, Weber F, Paludan SR, Hartmann R 2010 Extracellular 2′-5′ oligoadenylate synthetase stimulates RNase L-independent antiviral activity: a novel mechanism of virus-induced innate immunity. J Virol 84:11898–11904. doi:10.1128/JVI.01003-10. 20844035 PMC2977878

[19] Li LF, Yu J, Li Y, Wang J, Li S, Zhang L, Xia SL, Yang Q, Wang X, Yu S, Luo Y, Sun Y, Zhu Y, Munir M, Qiu HJ 2016 Guanylate-binding protein 1, an interferon-induced GTPase, exerts an antiviral activity against classical swine fever virus depending on its GTPase activity. J Virol 90:4412–4426. doi:10.1128/JVI.02718-15. 26889038 PMC4836331

[20] Hartmann R, Olsen HS, Widder S, Jorgensen R, Justesen J 1998 p59 OASL, a 2′-5′ oligoadenylate synthetase like protein: a novel human gene related to the 2′-5′ oligoadenylate synthetase family. Nucleic Acids Res 26:4121–4128. doi:10.1093/nar/26.18.4121. 9722630 PMC147837

[21] Rebouillat D, Marié I, Hovanessian AG 1998 Molecular cloning and characterization of two related and interferon-induced 56-kDa and 30-kDa proteins highly similar to 2′-5′ oligoadenylate synthetase. Eur J Biochem 257:319–330. doi:10.1046/j.1432-1327.1998.2570319.x. 9826176

[22] Kristiansen H, Gad HH, Eskildsen-Larsen S, Despres P, Hartmann R 2011 The oligoadenylate synthetase family: an ancient protein family with multiple antiviral activities. J Interferon Cytokine Res 31:41–47. doi:10.1089/jir.2010.0107. 21142819

[23] Itsui Y, Sakamoto N, Kurosaki M, Kanazawa N, Tanabe Y, Koyama T, Takeda Y, Nakagawa M, Kakinuma S, Sekine Y, Maekawa S, Enomoto N, Watanabe M 2006 Expressional screening of interferon-stimulated genes for antiviral activity against hepatitis C virus replication. J Viral Hepat 13:690–700. doi:10.1111/j.1365-2893.2006.00732.x. 16970601

[24] Pan W, Zuo X, Feng T, Shi X, Dai J 2012 Guanylate-binding protein 1 participates in cellular antiviral response to dengue virus. Virol J 9:292. doi:10.1186/1743-422X-9-292. 23186538 PMC3520834

[25] Dong XY, Liu WJ, Zhao MQ, Wang JY, Pei JJ, Luo YW, Ju CM, Chen JD 2013 Classical swine fever virus triggers RIG-I and MDA5-dependent signaling pathway to IRF-3 and NF-κB activation to promote secretion of interferon and inflammatory cytokines in porcine alveolar macrophages. Virol J 10:286. doi:10.1186/1743-422X-10-286. 24034559 PMC3849481

[26] Andersen JB, Li XL, Judge CS, Zhou A, Jha BK, Shelby S, Zhou L, Silverman RH, Hassel BA 2007 Role of 2-5A-dependent RNase-L in senescence and longevity. Oncogene 26:3081–3088. doi:10.1038/sj.onc.1210111. 17130839

[27] Justesen J, Hartmann R, Kjeldgaard N 2000 Gene structure and function of the 2′-5′ oligoadenylate synthetase family. Cell Mol Life Sci 57:1593–1612. doi:10.1007/PL00000644. 11092454 PMC11146851

[28] Mashimo T, Glaser P, Lucas M, Simon-Chazottes D, Ceccaldi PE, Montagutelli X, Desprès P, Guénet JL 2003 Structural and functional genomics and evolutionary relationships in the cluster of genes encoding murine 2′,5′-oligoadenylate synthetases. Genomics 82:537–552. doi:10.1016/S0888-7543(03)00176-9. 14559211

[29] Perelygin AA, Scherbik SV, Zhulin IB, Stockman BM, Li Y, Brinton MA 2002 Positional cloning of the murine flavivirus resistance gene. Proc Natl Acad Sci U S A 99:9322–9327. doi:10.1073/pnas.142287799. 12080145 PMC123139

[30] Kakuta S, Shibata S, Iwakura Y 2002 Genomic structure of the mouse 2′,5′-oligoadenylate synthetase gene family. J Interferon Cytokine Res 22:981–993. doi:10.1089/10799900260286696. 12396720

[31] Hovnanian A, Rebouillat D, Mattei MG, Levy ER, Marie I, Monaco AP, Hovanessian AG 1998 The human 2′,5′-oligoadenylate synthetase locus is composed of three distinct genes clustered on chromosome 12q24.2 encoding the 100-, 69-, and 40-kDa forms. Genomics 52:267–277. doi:10.1006/geno.1998.5443. 9790745

[32] Perelygin AA, Lear TL, Zharkikh AA, Brinton MA 2005 Structure of equine 2′-5′ oligoadenylate synthetase (Oas) gene family and FISH mapping of Oas genes to ECA8p15-p14 and BTA17q24-25. Cytogenet Genome Res 111:51–56. doi:10.1159/000085670. 16093721

[33] Zheng S, Zhu D, Lian X, Liu W, Cao R, Chen P 2016 Porcine 2′,5′-oligoadenylate synthetases inhibit Japanese encephalitis virus replication *in vitro*. J Med Virol 88:760–768. doi:10.1002/jmv.24397. 26437676

[34] Melchjorsen J, Kristiansen H, Christiansen R, Rintahaka J, Matikainen S, Paludan SR, Hartmann R 2009 Differential regulation of the OASL and OAS1 genes in response to viral infections. J Interferon Cytokine Res 29:199–207. doi:10.1089/jir.2008.0050. 19203244

[35] Talon J, Horvath CM, Polley R, Basler CF, Muster T, Palese P, García-Sastre A 2000 Activation of interferon regulatory factor 3 is inhibited by the influenza A virus NS1 protein. J Virol 74:7989–7996. doi:10.1128/JVI.74.17.7989-7996.2000. 10933707 PMC112330

[36] Tamura T, Nagashima N, Ruggli N, Summerfield A, Kida H, Sakoda Y 2014 N^pro^ of classical swine fever virus contributes to pathogenicity in pigs by preventing type I interferon induction at local replication sites. Vet Res 45:47. doi:10.1186/1297-9716-45-47. 24742209 PMC4018971

[37] Bauhofer O, Summerfield A, Sakoda Y, Tratschin JD, Hofmann MA, Ruggli N 2007 Classical swine fever virus N^pro^ interacts with interferon regulatory factor 3 and induces its proteasomal degradation. J Virol 81:3087–3096. doi:10.1128/JVI.02032-06. 17215286 PMC1866024

[38] Schoggins JW, Rice CM 2011 Interferon-stimulated genes and their antiviral effector functions. Curr Opin Virol 1:519–525. doi:10.1016/j.coviro.2011.10.008. 22328912 PMC3274382

[39] Choi UY, Kang JS, Hwang YS, Kim YJ 2015 Oligoadenylate synthase-like (OASL) proteins: dual functions and associations with diseases. Exp Mol Med 47:e144. doi:10.1038/emm.2014.110. 25744296 PMC4351405

[40] Lee M, Kim B, Oh GT, Kim YJ 2013 OASL1 inhibits translation of the type I interferon-regulating transcription factor IRF7. Nat Immunol 14:346–355. doi:10.1038/ni.2535. 23416614

[41] Schoggins JW, Wilson SJ, Panis M, Murphy MY, Jones CT, Bieniasz P, Rice CM 2011 A diverse range of gene products are effectors of the type I interferon antiviral response. Nature 472:481–485. doi:10.1038/nature09907. 21478870 PMC3409588

[42] Shen L, Li Y, Chen J, Li C, Huang J, Luo Y, Sun Y, Li S, Qiu HJ 2014 Generation of a recombinant classical swine fever virus stably expressing the firefly luciferase gene for quantitative antiviral assay. Antiviral Res 109:15–21. doi:10.1016/j.antiviral.2014.06.006. 24956495

[43] Reed LJ, Muench H 1938 A simple method of estimating 50 percent endpoints. Am J Hyg 27:493–497.

[44] Zhao JJ, Cheng D, Li N, Sun Y, Shi Z, Zhu QH, Tu C, Tong GZ, Qiu HJ 2008 Evaluation of a multiplex real-time RT-PCR for quantitative and differential detection of wild-type viruses and C-strain vaccine of classical swine fever virus. Vet Microbiol 126:1–10. doi:10.1016/j.vetmic.2007.04.046. 17658704

[45] Wang Y, Yuan J, Cong X, Qin HY, Wang CH, Li Y, Li S, Luo Y, Sun Y, Qiu HJ 2015 Generation and efficacy evaluation of a recombinant pseudorabies virus variant expressing the E2 protein of classical swine fever virus in pigs. Clin Vaccine Immunol 22:1121–1129. doi:10.1128/CVI.00383-15. 26311244 PMC4580741

